# Rifampicin Resistance: Fitness Costs and the Significance of Compensatory Evolution

**DOI:** 10.3390/antibiotics2020206

**Published:** 2013-04-03

**Authors:** Diarmaid Hughes, Gerrit Brandis

**Affiliations:** Department of Medical Biochemistry and Microbiology / Box 582, Biomedical Center, Uppsala University, Husargatan 3, Uppsala 75423, Sweden; E-Mail: gerrit.brandis@imbim.uu.se

**Keywords:** tuberculosis, *Salmonella*, genomics, genetics, combination therapy

## Abstract

Seventy years after the introduction of antibiotic chemotherapy to treat tuberculosis, problems caused by drug-resistance in *Mycobacterium tuberculosis* have become greater than ever. The discovery and development of novel drugs and drug combination therapies will be critical to managing these problematic infections. However, to maintain effective therapy in the long-term and to avoid repeating the mistakes of the past, it is essential that we understand how resistance to antibiotics evolves in *M. tuberculosis*. Recent studies in genomics and genetics, employing both clinical isolates and model organisms, have revealed that resistance to the frontline anti-tuberculosis drug, rifampicin, is very strongly associated with the selection of fitness compensatory mutations in the different subunits of RNA polymerase. This mode of resistance evolution may also apply to other drugs, and knowledge of the rates and mechanisms could be used to design improved diagnostics and by tracking the evolution of infectious strains, to inform the optimization of therapies.

## 1. Introduction

Tuberculosis, caused by infection of the airways and lungs by *Mycobacterium tuberculosis*, is a major cause of infectious disease-associated morbidity and mortality worldwide [1,2]. Treatment of tuberculosis is difficult and requires relatively long courses of antibiotic treatment. This leads to problems of resistance development during therapy, and as a result, effective therapy is only achieved through the use of combinations of antibiotics [3]. Over the years, new anti-tuberculosis drugs have been discovered, developed and tested, and this discovery process continues today [4], with several anti-tuberculosis drugs in clinical trials [5]. Ongoing drug development has also lead to changes and refinements in the recommended combinations of antibiotics and in therapeutic duration, to improve the effectiveness of therapy against drug-susceptible *M. tuberculosis* [3]. The increasing prevalence of multidrug-resistant tuberculosis in recent years, in part associated with HIV infections, is driving continuing efforts in drug discovery and development and testing of novel drug combinations, in an attempt to achieve more effective therapy against resistant infections [6]. While the development of new anti-tuberculosis drugs is critical for dealing with the immediate therapeutic problems, in the longer-term, it will also be very important to gain a better understanding of the evolutionary processes that drive drug resistance development, so that we have the possibility to develop rational approaches to reducing the problem of resistance [7].

Rifampicin is an oral rifamycin that was shown, in the 1960s, to be effective for treatment of tuberculosis [8,9]. Despite an increasing incidence of resistance, rifampicin remains an important antibiotic and is, together with isoniazid/ethambutol and pyrazinamide, an essential part of short-course anti-tuberculosis treatment [3,10]. The target of rifampicin is the β-subunit of the RNA polymerase, where it binds and inhibits the elongation of RNA transcription shortly after initiation [11,12]. Resistance to rifampicin arises due to single amino acid substitutions in the β-subunit. Classic studies in *E. coli* [13] supported by data from clinical isolates of *M. tuberculosis* [14] and from other organisms [15,16] show that rifampicin resistance is nearly always caused by any one of many different point mutations affecting a relatively small part of the β-subunit of RNA polymerase close to the catalytic center of the enzyme [12,17,18]. In *M. tuberculosis*, the clinical breakpoint for resistance to rifampicin has recently been revised downward, from a minimal inhibitory concentration (MIC) of 1.0 mg/L to 0.0625 mg/L, based on population pharmacokinetic studies [19,20]. In *E. coli* and *Salmonella*, where genetic studies are frequently carried out, rifampicin MIC for wild-type strains is approximately 12 mg/L, while a resistant mutant can have an MIC ≥3,000 mg/L [21].

## 2. Resistance and Fitness

**Figure 1:**
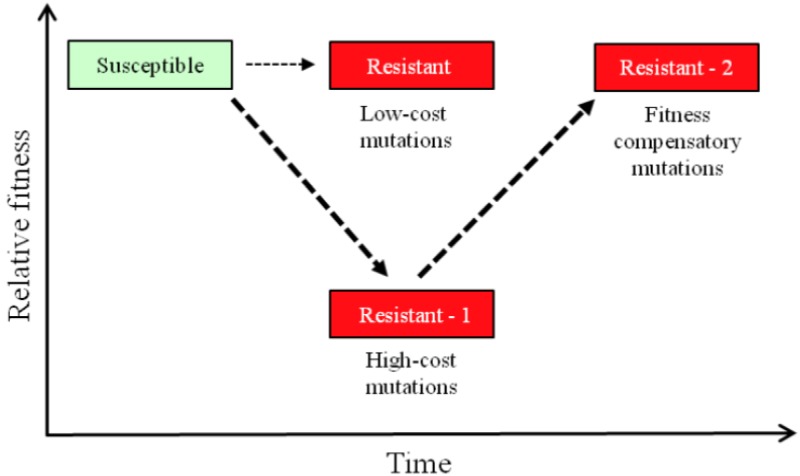
Evolution of resistance is usually a two-step process. Most frequently, resistance is initially associated with a reduction of relative fitness that can subsequently be ameliorated by acquisition and selection of additional fitness compensatory mutations.

The development of resistance to antibiotics in *M. tuberculosis* is associated with chromosomal mutations rather than with horizontal genetic transfer events, as is common in many other infectious bacteria [22]. Genetic and physiological studies in model organisms, like *Salmonella typhimurium* (*S. enterica* serovar Typhimurium) and *Escherichia coli*, have shown that most chromosomal mutations causing resistance to antibiotics are associated with significant fitness costs, both *in vitro* and *in vivo* [23,24,25]. The close association of significant fitness costs with antibiotic-resistance mutations is not surprising when one considers that the processes targeted by antibiotics are usually of central importance to bacterial growth and include the machinery of protein synthesis, DNA replication and transcription and the processes of cell wall synthesis [23]. The concept of a biological fitness cost usually refers to a measured reduction in relative bacterial growth rate in a particular environment, but the concept is broader and embraces for example reductions in transmission efficiency or changes in relative virulence in bacterial pathogens, including for *M. tuberculosis* [26]. The clinical significance of having fitness costs associated with resistance mutations is that it implies that in mixed bacterial populations and in the absence of sufficient antibiotic selective pressure, that antibiotic-resistant strains would be out-competed and progressively replaced by higher-fitness antibiotic-susceptible strains [25]. If that were the case, then it might be possible, in principle, to restrict the proportion of resistant strains in a population by restricting antibiotic use in general or by periodically cycling different antibiotics in clinical practice. However, there are several serious caveats to that hypothesis. Thus, experiments in model organisms, testing many different antibiotics and resistance mutations, have shown that secondary fitness-compensatory mutations arise frequently in resistant strains (Figure 1) and that these can significantly ameliorate the fitness costs of resistance [23,25]. Compensatory evolution can even make a bad situation worse. Thus, for fluoroquinolones, it has been shown in different species that some compensatory mutations that reduce the fitness costs of resistance mutations simultaneously increase the level of resistance to the antibiotic [27,28]. Accordingly, increased resistance can evolve by Darwinian selection for increased fitness even in the absence of continued antibiotic selection pressure. Also, experimental studies in *Pseudomonas aeruginosa* have shown that different deleterious rifampicin-resistance mutations can exhibit antagonistic epistasis, partially or completely compensating for each other’s fitness costs [29]. A second caveat concerns resistance mutations that are low-cost or cost-free (Figure 1). At sub-MIC antibiotic concentrations, a condition likely to be relevant for TB, where there is long-term treatment with multiple antibiotics, low-cost resistance is preferentially selected, and such mutants can outcompete the susceptible wild-type, even at very low antibiotic concentrations [30]. The prediction is that low-cost resistance mutations should be selectively enriched among resistant clinical strains, and there is some evidence that such enrichment occurs in *M. tuberculosis* [31,32,33]. A third complication, that may be particularly relevant for resistance arising in tuberculosis patients treated with combination therapy, is the possibility of epistatic interactions between mutations causing resistance to different antibiotics. It was shown, using *P. aeruginosa* as a model organism, that the fitness costs of individual mutations causing streptomycin-resistance varied significantly, depending on the presence of particular mutations causing resistance to rifampicin, and also on whether the antibiotic rifampicin was present or absent from the environment [34]. This conclusion, that epistatic interactions may play a role, is supported by a recent analysis of the relationships between resistance mutations and genetic background in multidrug-resistant isolates of *M. tuberculosis* [35]. Thus, epistasis and genotype-by-environment interactions may each have a significant influence the evolution of multidrug-resistance in *M. tuberculosis*. 

## 3. Measuring Fitness Costs

Ideally, the fitness costs of antibiotic resistance should be measured in defined groups of infected patients. However, for practical and ethical reasons, such an approach is, at least with current technologies, ruled out. Instead, fitness costs are usually measured in controlled laboratory experiments, using model environments and model organisms, as surrogates for the clinically relevant situations [23]. In measuring fitness, there are several aspects and parameters to consider. (i) It is critically important that isogenic strains be used for the comparison. This makes it practically difficult to use most clinical isolates, unless they can be genetically manipulated or selected, to generate an isogenic pair. In most cases, this problem is solved by selecting or generating mutations in laboratory strains that mimic the mutations found in clinical isolates. Thus, the direct clinical relevance of a particular genetic background usually has to be sacrificed in favor of making a genetically controlled experiment; (ii) It is important that the species being studied is amenable to genetic manipulation, so that individual mutations or combinations of mutations can be evaluated for their effects on resistance and fitness in standard or ‘wild-type’ genetic backgrounds, without the confounding effects of an uncontrolled number of additional genetic variations typically present in different clinical isolates. Such genetic manipulations have been made to determine the evolutionary path to aminoglycoside resistance in *M. smegmatis* [36], but they are so difficult to achieve in *M. tuberculosis*, that the use of more genetically amenable species is strongly favored; (iii) It is important that the model system used can be evaluated with a variety of different fitness assays, such that an assay with sufficient discriminatory power to differentiate between relevant isogenic variants can be chosen as appropriate [7,23]. Taking each of these factors into consideration, *S. typhimurium* represents a practical, genetically amenable, model system to measure the fitness costs associated with mutations to rifampicin resistance [21].

## 4. Resistance and Genomics Analysis

Rifampicin resistance is strongly correlated with mutations in a small stretch of DNA in the gene *rpoB*, which encodes the β-subunit of RNA polymerase. The so-called rifampicin-resistance-determining-region (RRDR) covers 81 base pairs encoding for the amino acids 507 to 533 in the β-subunit. The great majority (96%) of unrelated clinical rifampicin-resistant *M. tuberculosis* isolates were found to carry mutations or small deletions in this region that are absent in rifampicin-sensitive isolates [18]. That these mutations in the RNA polymerase are responsible for the rifampicin resistance was finally proven by the construction of mycobacterial shuttle plasmids containing different versions of the *rpoB* gene. Recombinant *Mycobacterium* isolates harboring a plasmid containing the wild-type *rpoB* gene showed no rifampicin resistance, while isolates that had a plasmid carrying a *rpoB* gene with a proposed Rif^R^ mutation were shown to have a rifampicin-resistance phenotype [37].

For the remaining 4% of isolates, no *rpoB* mutations were identified, neither within the RRDR of *rpoB* nor outside of it, which left open the possibility of mutations outside *rpoB* being associated with rifampicin resistance. Hypothesizing that efflux pumps might be responsible for the rifampicin-resistance phenotype in this 4% of isolates, transcriptional analyses of 20 efflux pump genes were performed on *M. tuberculosi*s isolates that were phenotypically resistant, but carried no mutation in *rpoB*. Three different efflux pumps (Rv2936, Rv0783 and Rv0933) were overexpressed in the resistant isolates, suggesting these pumps might be involved in reducing the intracellular rifampicin concentration of the cell. To further analyze whether overexpression of these efflux pumps conferred rifampicin resistance, the relevant pump genes were cloned onto expression vectors and moved into *E. coli* strains. Overexpression of Rv0783 caused a two-fold increase in rifampicin MIC, while overexpressing Rv2936 increased the rifampicin MIC four-fold. In contrast, overexpression of Rv0933 had no effect on rifampicin resistance. These data proved that overexpression of two different efflux pumps could generate a rifampicin-resistance phenotype in *M. tuberculosis* [38].

In addition to the mutations identified within the RRDR of *rpoB* that confer rifampicin resistance, two other regions of *rpoB* have been associated with low-level rifampicin-resistance in the *E. coli rpoB* gene. These are amino acids 148–153 and 1244–1260 [39]. The amino acids 148–153 lie within a 97 amino acid long insert in the *E. coli rpoB* gene that does not exist in the *M. tuberculosis* gene, and so far, no clinical isolates have been reported to carry any mutations in either of these regions, suggesting that these mutations are not of clinical importance.

**Figure 2:**
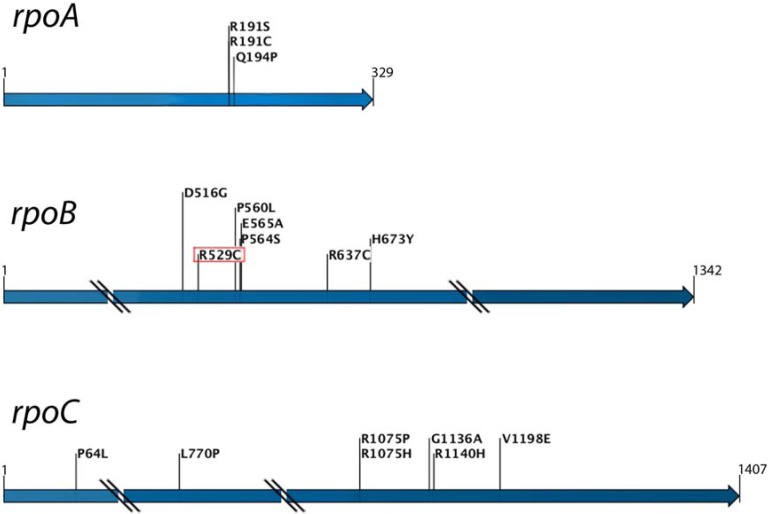
Fitness compensatory mutations that ameliorate the fitness costs of the rifampicin-resistance mutation, *rpoB* R529C, occur in *rpoA*, *rpoB* and *rpoC* genes, coding for different subunits of RNA polymerase.

As with many other resistance mutations, Rif^R^ mutations do not come without a cost. Competing laboratory-derived rifampicin-resistant isolates against the rifampicin-sensitive parental strain shows a competitive disadvantage of the Rif^R^ mutation in the absence of the drug. Depending on the particular mutation, the strain genetic background and the type of competition assay, the fitness of Rif^R^ mutants has been found to vary from being indistinguishable from the susceptible wild-type strains down to a relative fitness of approximately 0.2 [33,40,41]. In general, resistance mutations with a lower measured fitness cost in *in vitro* assays are also the resistance mutations that are more frequently identified in clinical isolates. However, it is notable that resistant clinical isolates have been found to have lower or even no fitness cost, compared with the costs associated with the same resistance mutations measured *in vitro*-derived isolates. Competition of clinically-derived rifampicin-resistant isolates against their drug-susceptible ancestors indicates that the fitness cost of Rif^R^ mutations is generally lower the longer the resistant strain has infected the patient, suggesting that fitness-compensatory evolution may have occurred *in vivo* [40]. Genetic evidence for compensatory evolution to reduce the fitness costs associated with rifampicin-resistant RNA polymerase was first shown in *E. coli*. Four strains with different Rif^R^ mutations were evolved *in vitro* by serial passage for increased competiveness fitness. After 200 generations of evolution, isolates of three of the four strains showed a significant increase in growth rate, proving that compensatory evolution had taken place. Partial sequencing of the *rpoB* gene of the evolved isolates showed secondary mutations within the gene in about half the isolates, suggesting that secondary mutations within the *rpoB* gene can compensate the fitness cost of the initial Rif^R^ mutation [42]. Since clinical *M. tuberculosis* isolates sometimes contain multiple mutations within the RRDR of *rpoB* and the majority of the putative compensatory mutations in *E. coli* were found within this region, it is reasonable to conclude that the presence of multiple mutations in clinical isolates also indicates a mixture of primary Rif^R^ mutations and secondary compensatory mutations. Even so, in about half of the evolved *E. coli* strains, no compensatory mutation was identified within the *rpoB* gene, suggesting that the fitness cost caused by the primary Rif^R^ mutation could also be compensated by mutations outside this gene. In a more recent study, a *S. typhimurium* strain containing the high-cost Rif^R^ mutation *rpoB* R529C was evolved for 60 generations by serial passage to select increased fitness. Faster growing isolates were identified, and the genes, *rpoA*, *rpoB* and *rpoC*, encoding for the RNAP α, β and β' subunits, respectively, were sequenced. In agreement with the previous *E. coli* data, about half of the strains were found to contain a secondary mutation within *rpoB*. In the other strains, secondary mutations were found in *rpoA* or *rpoC* (Figure 2). With the exception of one secondary mutation in *rpoB* that by itself causes a Rif^R^ phenotype, the secondary mutations selected in *E.* coli and *Salmonella* did not significantly increase or decrease the MIC [21,42]. Genetic reconstructions were made and showed that each of these secondary mutations was necessary and sufficient to compensate for the fitness cost caused by the primary Rif^R^ mutation, proving that mutations within three different subunits of RNA polymerase can compensate for the fitness cost caused by a primary Rif^R^ mutation [21]. The reasons why fitness costs are frequently associated with Rif^R^ mutations are not fully understood. However, at least some mutations in *rpoB* destabilize the interaction between RNAP and ribosomal RNA promoters in *E. coli*, a phenotype that could explain reduced fitness and also could plausibly be compensated by secondary mutations affecting other subunits of RNAP [43,44]. 

Recent studies on rifampicin-resistant *M. tuberculosis* have underlined the clinical importance of these compensatory mutations. Whole genome sequencing of ten paired clinical rifampicin-resistant isolates and their susceptible ancestors showed that four of the ten isolates had a putative compensatory mutation in the *rpoA* or *rpoC* gene. Local sequencing of the *rpoA*, *rpoB* and a part of the *rpoC* genes from 117 MDR strains representing the five main global MTBC lineages and 212 MDR strains from high-burden countries (Abkhazia/Georgia, Kazakhstan and Uzbekistan) showed that about 20% of the global and 32% of the high-burden MTBC isolates, contained a secondary mutation in one of the three RNAP genes [45]. Another study analyzing whole genome sequences of *M. tuberculosis* strains of the Beijing family isolated in Russia further confirmed these results. Twenty-six rifampicin-resistant and seven susceptible strains were analyzed. While none of the susceptible strains carried a mutation in any of the RNAP genes, except one mutation in *rpoC* that arose before the evolution of rifampicin-resistance, each one of the 26 Rif^R^ strains had at least one known Rif^R^ mutation in *rpoB* and at least one additional mutation in *rpoA*, *rpoB* or *rpoC* [46]. These studies show that secondary mutations in the RNA polymerase genes occur frequently, and based on the conclusions from the genetic reconstructions made in *Salmonella* [21], they presumably compensate for the fitness costs of primary Rif^R^ mutations in clinical *M. tuberculosis*. Another study of compensatory mutations in the *rpoC* gene of rifampicin-resistant *M. tuberculosis* suggests that these secondary mutations may increase not only the fitness of the Rif^R^ strains, but may also enhance the spread of the resistant strain. A set of 286 drug-resistant and 54 drug-sensitive clinical isolates from Cape Town, South Africa, were analyzed for the presence of mutations in a small part of the *rpoC* gene, which had earlier been found to be a hot spot for putative compensatory mutations [45] and classified according to their IS*6110* RFLP patterns. Interestingly, putative compensatory mutations in *rpoC* were found in 31% of the isolates belonging to recognized RFLP clusters, while only 9% of the isolates with non-clustered RFLP patterns harbored secondary mutations in *rpoC*. Since clustered RFLP patterns are associated with ongoing transmission, the data suggest that rifampicin-resistant strains with a secondary mutation in *rpoC* are more likely to spread within the population than strains without a secondary mutation [47].

## 5. Conclusions

The data from genomics and genetics analyses support the conclusion that the evolution of rifampicin-resistance in *M. tuberculosis* is strongly associated with selection for fitness-compensatory mutations, occurring in different subunits of the RNA polymerase. This knowledge could be applied to study the evolution of resistance to other drugs and in the design of improved diagnostics. By tracking the evolutionary trajectories of infectious strains, it may be possible to use genomics information as an aid in the optimization of tuberculosis therapies.
